# Circular RNA circCCDC9 alleviates ischaemic stroke ischaemia/reperfusion injury via the Notch pathway

**DOI:** 10.1111/jcmm.16025

**Published:** 2020-10-29

**Authors:** Liquan Wu, Haitao Xu, Wenfei Zhang, Zhibiao Chen, Wenlan Li, Wei Ke

**Affiliations:** ^1^ Department of Neurosurgery Renmin Hospital of Wuhan University Wuhan China; ^2^ Department of Anesthesiology Renmin Hospital of Wuhan University Wuhan China; ^3^ Department of Neurology Renmin Hospital of Wuhan University Wuhan China

**Keywords:** cerebral ischaemia/reperfusion, circCCDC9, Notch pathway

## Abstract

Stroke is a leading cause of death and disability, while its pathophysiological mechanisms are not fully understood. In this study, we used the tMCAO mice model to investigate the role of circCCDC9 in the pathogenesis of stroke. We found that the expression of circCCDC9 was significantly decreased in the brains of tMCAO mice. The Evens blue and brain water content were significantly higher in the Pre‐IR and Pre‐IR+Vector mice, while these patterns were partially reversed by overexpression of circCCDC9. The nitrite content and eNOS expression were decreased in the Pre‐IR and Pre‐IR+Vector groups, which was restored by circCCDC9 overexpression. Overexpression of circCCDC9 also inhibited the expression of Caspase‐3, Bax/Bcl‐2 ratio and the expression of Notch1, NICD and Hes1 in tMCAO mice. Knockdown of circCCDC9 increased the expression of Caspase‐3, Bax/Bcl‐2 ratio and the expression of Notch1, NICD and Hes1. In summary, overexpression of circCCDC9 protected the blood‐brain barrier and inhibited apoptosis by suppressing the Notch1 signalling pathway, while knockdown of circCCDC9 had the opposite effects. Our findings showed that circCCDC9 is a potential novel therapeutic target for cerebrovascular protection in acute ischaemic stroke.

## 1 INTRODUCTION

Stroke is a leading cause of mortality and disability globally.^1^, ^2^, ^3^ There are more than two million ischaemic stroke cases in adults aged 18‐50 years annually.^3^ Modifiable lifestyle risk factors, including smoking, physical activity, diet, alcohol consumption and weight, could cut down the risk of stroke. For patients with ischaemic stroke, the most effective and frequently used treatment would be early reperfusion with intravenous thrombolysis and thrombectomy. However, these treatments are limited by a narrow time window and side effects.^4^ Given this situation, there is an urgent need to develop novel treatments for patients with ischaemic stroke.

Circular RNAs are a class of non‐coding RNAs that are involved in the development of vascular diseases.^5^, ^6^ Circular circTLK1 deteriorated myocardial ischaemia/reperfusion injury via regulating the TNF signalling pathway.^7^ CircRNAs are abundant in the brain tissues^8^ and have been implicated in neurological functions^9^ and acute ischaemic stroke.^6^ A total of 521 differentially expressed circRNAs were found in the peripheral blood mononuclear cells of patients with acute ischaemic stroke compared to healthy controls, which suggested that circRNAs were potential markers for the diagnosis and treatment of ischaemic stroke.^10^ Using a more sophisticated study design, researchers found that the expression levels of circFUNDC1, circPDS5B and circCDC14A were increased and were positively correlated with infarct size after acute ischaemic stroke.^11^ These up‐regulated circRNAs also seemed to originate from different blood cells.^11^ It was found that circSCMH1 promoted the neuronal plasticity and inhibited glial cell activation and peripheral immune cell infiltration after stroke.^12^ The silencing of circHIPK2 promoted the differentiation of neural stem cells to neurons in vitro.^13^ Nevertheless, the roles of circRNAs in the physiological and neurological functions after ischaemic stroke remain mostly unexplored.

Overexpression of circCCDC9 was shown to inhibit the proliferation, migration and invasion of gastric cancer cells.^14^ However, its role in ischaemic stroke was unexplored. Here, we measured the expression of circCCDC9 in tMCAO mice. We further investigated the roles of circCCDC9 in regulating the cerebral I/R injury by assessing the brain function, cell apoptosis, and the expression of Notch1 and Hes1. Our results indicated that circCCDC9 is a potential therapeutic target for ischaemic stroke.

## 2 METHODS

### 2.1 Animals

Male C57BL/6J mice (25‐30 g, 8‐10 weeks) were from the Model Animal Center of Nanjing University. All mice were housed at 20°C with a 12 hours light‐dark cycle and ad libitum access to food and water. All animal work was approved by the Animal Care and Use Committee of the Renmin Hospital of Wuhan University.

### 2.2 tMCAO

The transient middle cerebral artery occlusion (tMCAO) was performed as previously described.^15^ Briefly, anaesthesia was induced with 4% chloral hydrate, and the mice were maintained at 37°C. The left internal and external carotid arteries were carefully exposed. A 6‐0 nylon filament was introduced into the left external carotid artery and slowly pushed into the internal carotid artery to block the blood flow of the middle cerebral artery. After 90 minutes of occlusion, the filament was gently removed to allow reperfusion. The sham mice were prepared for tMCAO but without the insertion.

### 2.3 Neurological scores

To determine the neurological deficits after tMCAO, we randomly divided twelve mice into the sham group and I/R (Pre‐IR) group as previously described.^16^ The mice were scored on a scale of 0‐4 at 24, 48 and 72 hours after reperfusion. Higher scores indicated severer neurological deficits. Points were defined as follows: 0, no deficit; 1, forelimb weakness; 2, circling to affected side; 3, partial paralysis on affected side; and 4, no spontaneous motor activity. The examiners were blinded to the treatment group.

### 2.4 Transfection

Mice were randomly assigned to each group (twenty per group): sham, Pre‐IR, Pre‐IR+Vector, Pre‐IR+OE‐circ, Pre‐IR+si‐NC and Pre‐IR+si‐circ. The lentivirus vectors and lentivirus carrying OE‐circ or siRNA were from the GenePharma Corp., Ltd. The mice were anaesthetized, and lentivirus was administered into the right intracerebroventricular slowly. The tMCAO surgery was performed at 14 days after injection.

### 2.5 TTC staining

The infarct volume was assessed at 24 following tMCAO. Five mice of each group were anaesthetized and decapitated. The brains were collected, fixed in 10% formaldehyde for 24 hours and coronally sectioned into 2‐mm slices. The slices were stained with 2% TTC (2,3,5‐triphenyl tetrazolium chloride, Sigma‐Aldrich) at 37°C for 10 minutes. The infarct areas were measured using ImageJ. The volume of infarcted brains was calculated by integrating the infarct areas of all brain slices.

### 2.6 Blood‐brain barrier integrity assays

Evans blue solution (Sigma‐Aldrich) was injected into the jugular vein of mice to allow 24 hours of circulation. Then, the brains were harvested and homogenized with PBS and centrifuged at 10 000 *g* for 30 minutes. The concentration of Evans blue was measured by a spectrophotometer (Thermo Fisher Scientific, Inc) at 610 nm. The brain water content was measured by drying mice brains to constant weight at 120°C. Nitric oxide release was measured using the NO assay kit (Beyotime) in line with the instructions. Five mice brains from each group were homogenized, centrifuged, and the supernatant was mixed with Griess reagents. The nitrite concentration was measured using a spectrophotometer at 540 nm.

### 2.7 TUNEL staining

The cell apoptosis was assessed using a TUNEL staining kit (Roche). Brain slices were embedded in 4% paraformaldehyde for 1, rehydrated with gradient alcohol, digested with Proteinase K (Sigma‐Aldrich) at room temperature for 30 minutes and incubated with the TUNEL mixture at 37°C for1 hour. Finally, the slices were counterstained with DAPI for five min. The percentage of apoptotic cells was calculated counted in 5 random fields under a microscope.

### 2.8 Real‐time polymerase chain reaction

Total RNA was extracted from brain tissues using the PureLink RNA Mini Kit (Invitrogen, Thermo Fisher Scientific). Complementary DNA (cDNA) was prepared using the PrimeScript™ RT Reagent Kit (9109, Takara). The PCR was carried out using the SYBR Green Master Mix (A25741, Applied Biosystems). The expression of target RNAs was normalized to GAPDH and calculated using the 2^−DΔCt^ method.

### 2.9 Western blot analysis

Total protein was extracted with Pierce IP Lysis Buffer (Thermo Fisher Scientific) and was quantified using the Pierce BCA Protein Assay Kit (Thermo Scientific). Aliquots of protein (50 μg) were separated by SDS‐PAGE on 10% gels and were transferred to polyvinylidene fluoride membranes. The membranes were then blocked with 5% skimmed milk at room temperature for two h. After blocking, the membranes were incubated overnight with: Anti‑endothelial NO synthase (eNOS), anti‐Bax, anti‐Bcl‐2, anti‐Notch1 (3608, 1:1000), anti‐NICD (8925, 1:1000) and anti‐Hes1 (11988, 1:1000) (Cell Signaling Technology). Following incubation, the membranes were probed with horseradish peroxidase‐conjugated secondary antibodies (Cell Signaling) at room temperature for 1 hour. The bands were visualized using the Enhanced Chemiluminescence Kit (GE Healthcare) and analysed using ImageJ.

### 2.10 Statistical analysis

Statistical analyses were performed using SPSS 22.0 (SPSS, IBM, USA). The results were described as means and standard deviations (SD). Student's *t* test was used to test the differences between groups. *P* < .05 indicates statistical significance.

## 3 RESULTS

### 3.1 Expression of circCCDC9 expression was down‐regulated after cerebral I/R injury in mice

**FIGURE 1:**
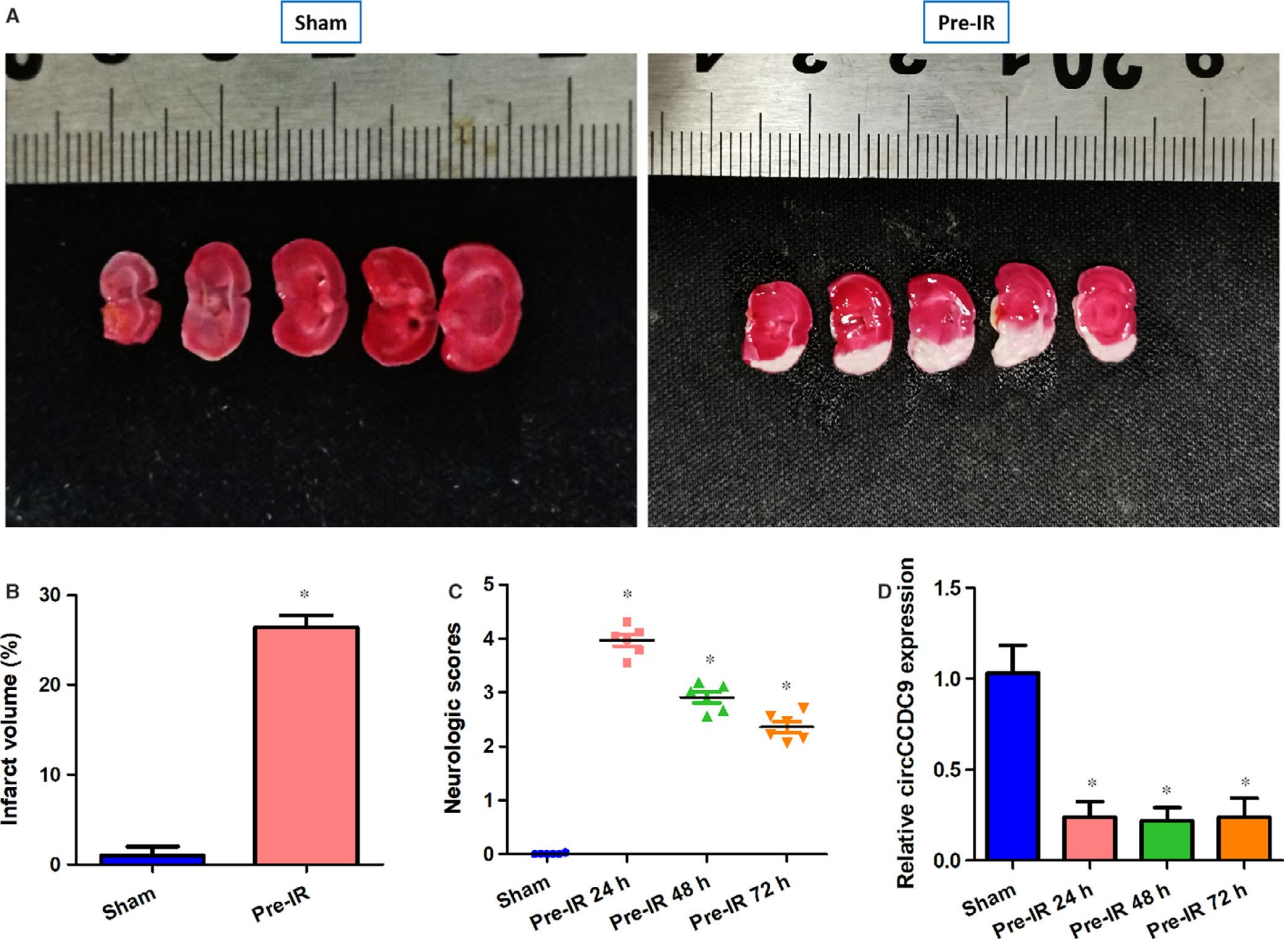
Effects of tMCAO in mice. (A & B) Brain infarct indicated using TTC staining 24 h after tMCAO (n = 5). (B) Neurological scores evaluated at 24, 48 and 72 h post‐tMCAO (n = 6). 0, no deficit; 1, forelimb weakness; 2, circling to affected side; 3, partial paralysis on affected side; and 4, no spontaneous motor activity. (D) Expression of circCCDC9 was measured at 24, 48 and 72 h post tMCAO (n = 5). Data are shown as means ± SD. *P < .05

We first assessed the levels of brain damage and the relative expression of circCCDC9 in the mouse model of ischaemia/reperfusion injury. Figure 1A,B show the effects of MCAO‐induced brain infarct in mice with TTC staining. The sham groups showed little sign of infarct of the brains while the Pre‐IR group had a significantly higher infarct volume. We used the neurological score to indicate the severity of brain damage. Higher scores indicated severer neurological deficits. Compared with mice in the sham group, the Pre‐IR mice had a higher neurological score at 24 hours post‐operation, which decreased with time until 72 hours (Figure 1C). The expression of circ_ circCCDC9 decreased significantly at 24 hours after MCAO and remain stable until 72 hours as shown in Figure 1D.

### 3.2 Overexpression of circCCDC9 protected the blood‐brain barrier after cerebral I/R injury in mice

**FIGURE 2:**
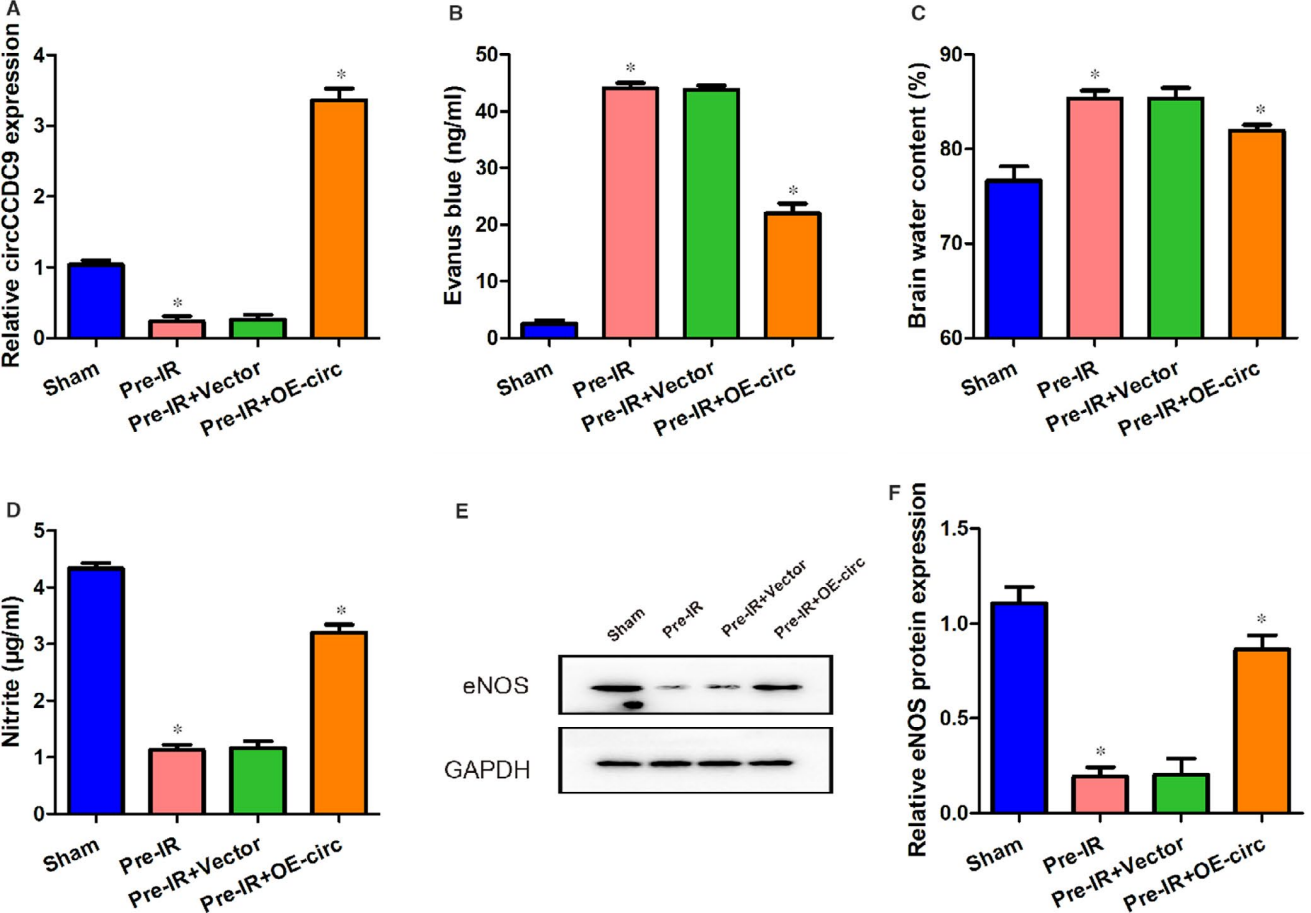
Overexpression of circCCDC9 protected the blood‐brain barrier after cerebral I/R injury in mice. (A) Expression of circCCDC9 in four groups at 24 h after tMCAO. Evans blue (B), brain water content (C) and nitrite (D) were measured at 24 h after tMCAO. (E, F) Protein expression of eNOS was assessed using Western blot at 24 h after tMCAO. Data are shown as means ± SD. *P < .05

In order to investigate if overexpression of circCCDC9 may be involved in modulating the function of the blood‐brain barrier after cerebral I/R injury, we overexpressed circCCDC9 and measured the levels of Evenus blue, brain water content, nitrite and expression of endothelial NO synthase (eNOS). The Evens blue staining and brain water content were measured to determine the effects of circCCDC9 on blood‐brain barrier permeability. eNOS and catalyses the amino acid L‑arginine conversion to NO, thus maintaining endovascular homoeostasis.^17^ The expression of circCCDC9 was successfully overexpressed in the Pre‐IR+OE‐circ group (Figure 2A). We found that the Evens blue and brain water content measurements were significantly higher in the Pre‐IR and Pre‐IR+Vector mice, while these patterns were partially reversed by overexpression of circCCDC9 (Figure 2B,C). On the contrary, the nitrite content decreased in the Pre‐IR and Pre‐IR+Vector groups, which was restored by circCCDC9 overexpression (Figure 2D). Similarly, the expression (eNOS) decreased significantly in the Pre‐IR and Pre‐IR+Vector groups, which was restored by circCCDC9 overexpression (Figure 2D,E). These results indicated that overexpression of circCCDC9 improved the function of the blood‐brain barrier after cerebral I/R injury in mice.

### 3.3 Overexpression of circCCDC9 inhibited apoptosis after cerebral I/R injury in mice

**FIGURE 3:**
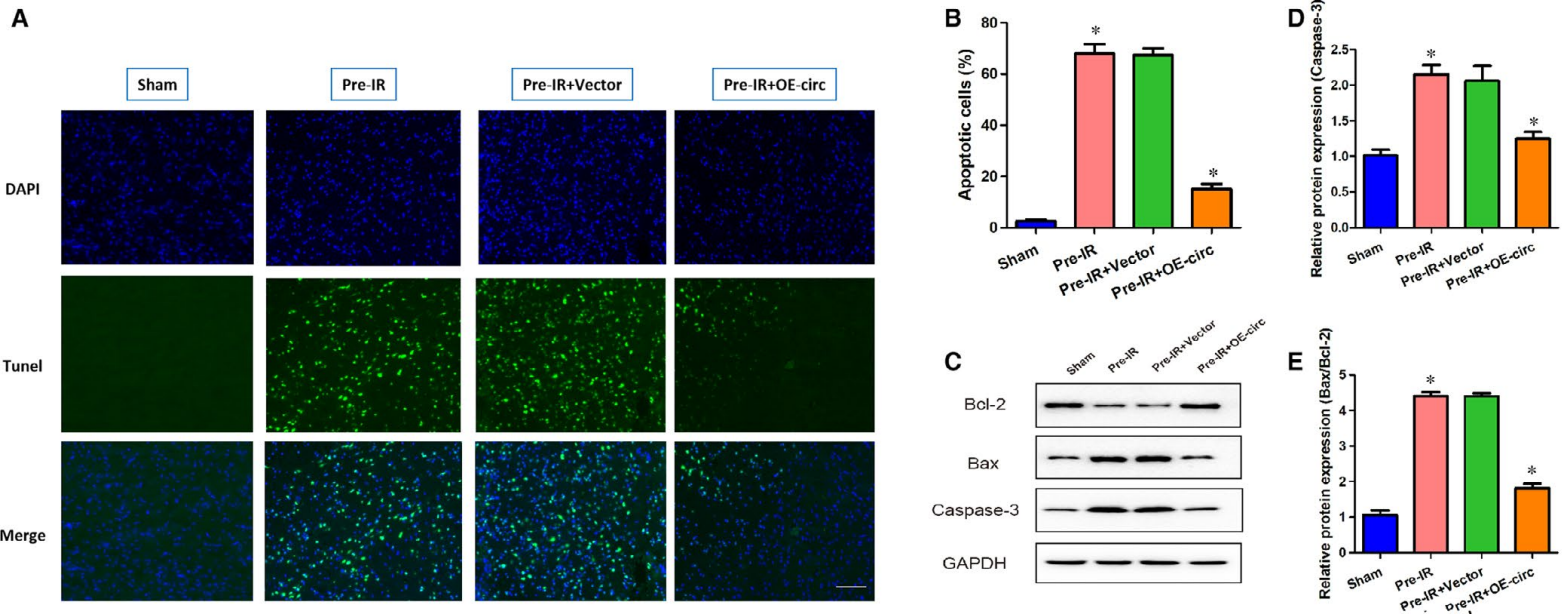
Overexpression of circCCDC9 inhibited apoptosis after cerebral I/R injury in mice. (A, B) Cerebral cell apoptosis was determined by TUNEL staining. (C‐E) The expressions of Bax, Bcl‐2 and Caspase‐3 proteins in mice were determined by Western blot at 24 h after tMCAO. Data are shown as means ± SD. *P < .05

Cerebral I/R injury leads to BBB disruption, which may cause neuronal apoptosis and neurological damage.^18^ Therefore, we next used TUNEL staining to assess whether circCCDC9 is involved in the regulation of neuronal apoptosis after cerebral I/R injury. The percentages of apoptotic cells were significantly increased in the Pre‐IR and Pre‐IR+Vector mice, while this trend was partially restored in mice transfected with OE‐circ (Figure 3A,B). The expressions of apoptosis markers were measured using Western blot. The ratio of Bax/Bcl‐2 and expression of Caspase‐3 were significantly increased in the Pre‐IR and Pre‐IR+Vector mice, which was ameliorated by circCCDC9 overexpression (Figure 3D). These results indicated that overexpression of circCCDC9 inhibited the apoptosis after cerebral I/R injury in mice.

### 3.4 Overexpression of circCCDC9 inhibited the expression of Notch1, NICD and Hes1 after cerebral I/R injury in mice

**FIGURE 4:**
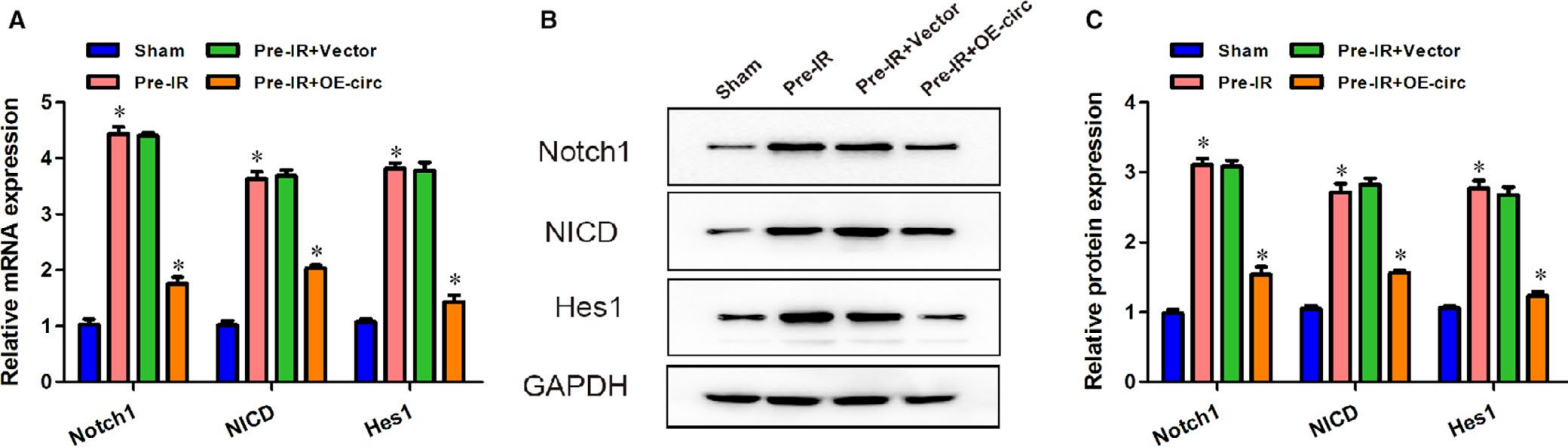
Overexpression of circCCDC9 inhibited the Notch pathway after cerebral I/R injury in mice. (A) The mRNA expressions of Notch1, NICD and Hes1 at 24 h after tMCAO. (B, C) Protein expressions of Notch1, NICD and Hes1 measured at 24 h after tMCAO. Data are shown as means ± SD. *P < .05

Previous studies have reported that the Notch1 expression was significantly increased in cerebral ischaemia.^19^ Activation of the Notch pathway may harm neurons in ischaemic stroke by increasing vulnerability to apoptosis. To determine whether circCCDC9 functions via the Notch pathway, we measured the expressions of Notch1, NICD and Hes1 at 24 hours following tMCAO. The mRNA expressions of Notch1, NICD and Hes1 were significantly increased in mice after tMCAO procedures, which were inhibited by the overexpression of circCCDC9 (Figure 4A). The protein expression of Notch1, NICD and Hes1 showed similar patterns in the four groups (Figure 4B,C). These results indicated that the Notch pathway was activated after cerebral I/R injury in mice, which was suppressed by overexpression of circCCDC9.

### 3.5 Knockdown of circCCDC9 endangered the blood‐brain barrier after cerebral I/R injury in mice

**FIGURE 5:**
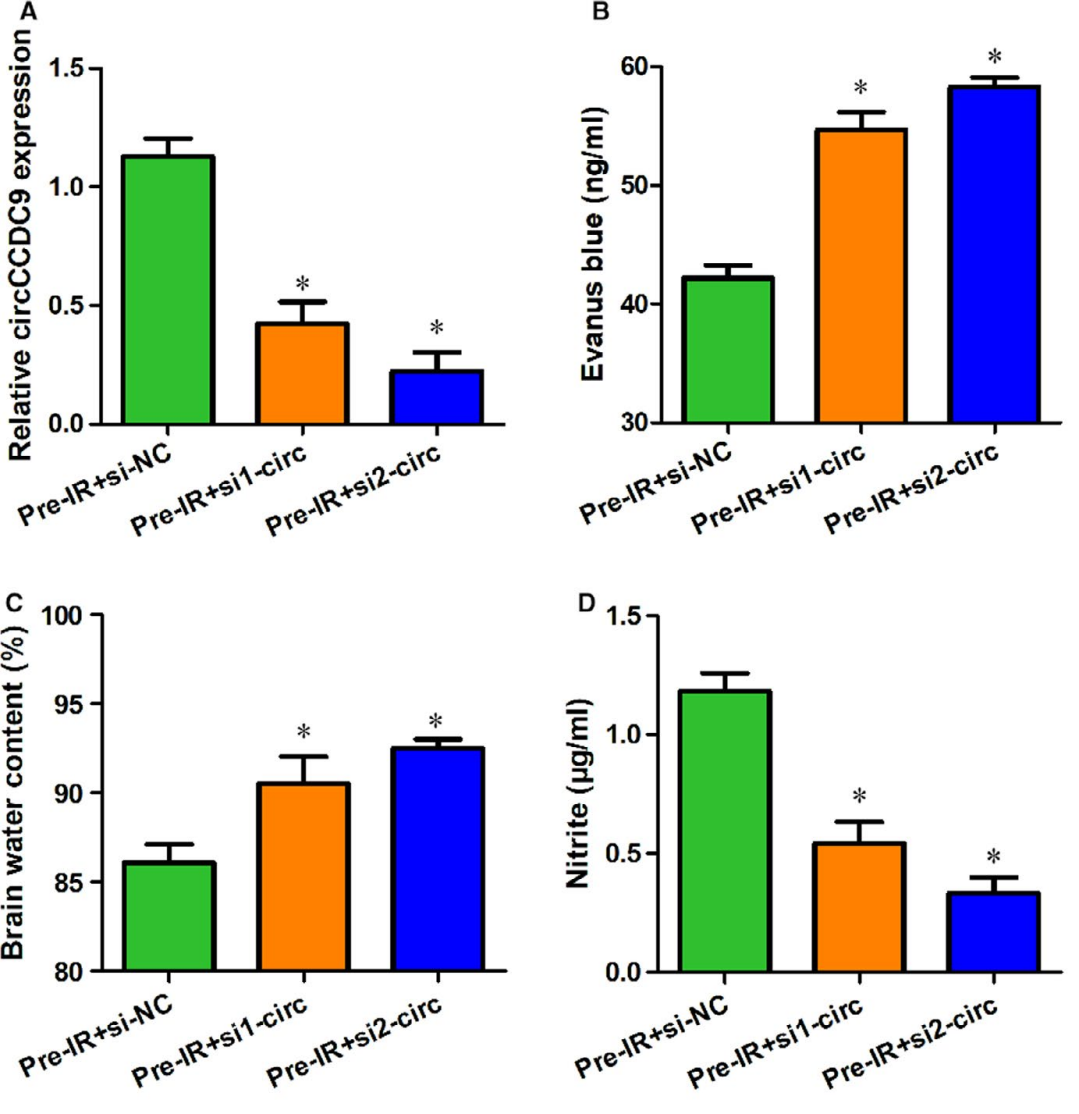
Knockdown of circCCDC9 endangered the blood‐brain barrier after cerebral I/R injury in mice. (A) Expression of circCCDC9 was measured in mice transfected with Pre‐IR+si‐NC, Pre‐IR+si1‐circ and Pre‐IR+si2‐ circ at 24 h after tMCAO. Evans blue (B), brain water content (C) and nitrite (D) were measured at 24 h after MCAO. Data are shown as means ± SD. *P < .05

Our results have shown that overexpression of circCCDC9 protected the blood‐brain barrier after cerebral I/R injury. Then, we investigated the effects of circCCDC9 knockdown using two siRNA against circCCDC9. The expression of circCCDC9 was successfully knocked down by si1‐circ and si2‐circ (Figure 5A). We found that the levels of Evens blue and brain water content were significantly increased in the si1‐circ and si2‐circ groups (Figure 5B,C). The nitrite content was decreased in the si1‐circ and si2‐circ groups (Figure 5D). These results indicated that knockdown of circCCDC9 impaired the blood‐brain barrier after cerebral I/R injury in mice.

### 3.6 Knockdown of circCCDC9 promoted apoptosis and inhibited the expression of Notch1, NICD and Hes1 after cerebral I/R injury in mice

**FIGURE 6:**
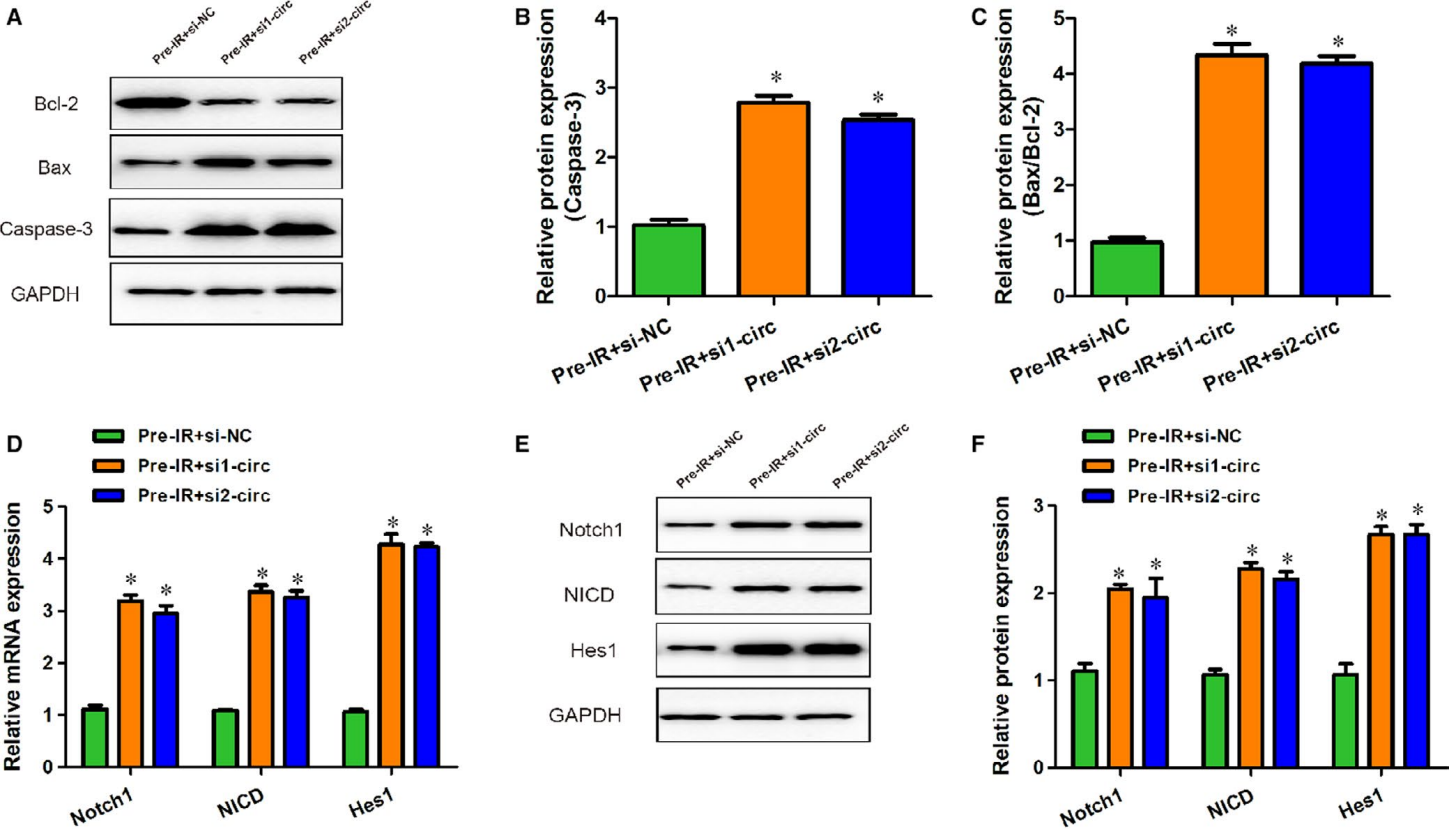
Knockdown of circCCDC9 promoted apoptosis after cerebral I/R injury in mice. (A‐C) The expressions of Bax and, Bcl‐2 and Caspase‐3 proteins in mice were determined by Western blot at 24 h after tMCAO. (D) The mRNA expressions of Notch1, NICD and Hes1 at 24 h after tMCAO. (E, F) Protein expressions of Notch1, NICD and Hes1 measured at 24 h after tMCAO. Data are shown as means ± SD. *P < .05

To determine whether circCCDC9 knockdown may have the opposite effects of overexpression on cell apoptosis, we measured the expressions of apoptosis markers in mice transfected with siRNA against circCCDC9. We found that the expression of Caspase‐3 and Bax/Bcl‐2 ratio was significantly increased in the circCCDC9 knockdown (Figure 6A‐C). We then assessed the expressions of Notch1, NICD and Hes1 after circCCDC9 knockdown. We found that the mRNA expressions of Notch1, NICD and Hes1 were significantly increased in the circCCDC9 knockdown groups (Figure 6D). Besides, the protein expression of Notch1, NICD and Hes1 was also increased (Figure 6E,F). These results indicated that circCCDC9 knockdown promoted apoptosis via activation of the Notch pathway.

## 4 DISCUSSION

Circular RNAs have been reported to be involved in a variety of human nervous system diseases, including Alzheimer's disease,^20^ Schizophrenia and depression,^21^ spinal cord injury^22^ and ischaemic stroke.^12^ A previous study found that circRNAs were differentially expressed during the differentiation of mouse neural stem cells, suggesting that circRNAs may be involved in the regulation of neural stem celldifferentiation.^23^ In vitro experiments have confirmed that CircHIPK2 silencing promoted the directional differentiation of neural stem cells into neurons but not into astrocytes and improved the neuronal plasticity.^13^ Yang and colleagues demonstrated an RNA drug delivery system that delivered circSCMH1, a significantly reduced circRNA in the plasma of acute ischaemic stroke patients, to the brain, which improved the functional recovery in both mice and monkey models of stroke.^12^ Our study focused on the role of circCCDC9 in the pathogenesis and development of stroke while the other circular RNAs were not measured. We found that circCCDC9 was decreased significantly after reperfusion and remained at a low level in the brain of tMCAO mice. It can thus be reasonable to assume that circCCDC9 plays a part in the regulation of cerebral injury after acute ischaemic stroke.

It has been shown that stroke influenced the expression of circRNAs in the brains of tMCAO mice.^24^ As the blood‐brain barrier was impaired during acute ischaemic stroke, differentially expressed circRNAs enter the bloodstream and thereby can be used as biomarkers for evaluating the progression of stroke. A study profiling the expression of circRNAs in the blood of mice with induced focal ischaemic stroke suggested that circPHKA2 and circBBS2 were promising markers for diagnosing acute ischaemic stroke.^25^ Plasma circDLGAP4 is thought to be a promising biomarker for the diagnosis and evaluation of ischaemic brain damage.^13^ Consistent with previous studies, the brain water and Evans blue content were significantly increased after the tMCAO procedures, indicating leakage of the blood‐brain barrier. It is worth noting that the leakage of the blood‐brain barrier was partially restored by the overexpression of circCCDC9 and was worsened by circCCDC9 knockdown.

To determine the mechanism of the protective role of circCCDC9 on the blood‐brain barrier, we compared the production of NO and NO synthase between groups. The endothelial NO synthase (eNO) produces NO that contributes to the protection of endothelial homoeostasis and enhancing the blood flow.^26^ In our study, the NO and eNO expression were significantly lower in the tMCAO mice, which were reversed by circCCDC9 overexpression. Notably, the apoptotic cells were also markedly reduced by circCCDC9 overexpression. These results suggested that the overexpression of circCCDC9 improved the integrity of the blood‐brain barrier by regulating the expression of eNO.

It has been documented that the Notch signalling pathway is involved in the pathophysiology of ischaemic stroke.^27^ Notch is an important membrane receptor that regulates the proliferation, differentiation and apoptosis of neurons.^28^ Notch signalling may promote the survival of neural stem cells during the recovery and remodelling stages after ischaemic stroke.^29^, ^30^ However, when ischaemia is more severe the Notch signalling induces neuronal death by interacting with other pathways, including NF‐κB, p53, HIF‐1α and Pin1.^28^ Neuronal cell apoptosis could be reduced by blocking the Notch signalling pathway, thus improving the prognosis.^31^ We have been suggested that circCCDC9 might play a role in cerebral I/R injury via the Notch pathway. In our study, the Notch pathway was activated in the tMCAO mice. Moreover, the expressions of Notch1, NICD and Hes1 were inhibited by circCCDC9 overexpression but were enhanced by circCCDC9 knockdown. These data confirmed that circCCDC9 regulated the Notch signalling pathway in I/R injury.

In summary, this study explored the potential effects of circCCDC9 on acute ischaemic stroke using the tMCAO mouse model. Overexpression of circCCDC9 improved the leakage of the blood‐brain barrier and inhibited the apoptosis of neurons, while knockdown of circCCDC9 had the opposite effects. Furthermore, circCCDC9 played a crucial role in regulating the Notch signalling pathway. These findings suggest that circCCDC9 may be a novel therapeutic target for ischaemic stroke.

## CONFLICT OF INTEREST

The authors declare that they have no conflict of interest.

## AUTHOR CONTRIBUTIONS

**Liquan Wu:** Conceptualization (equal); project administration (equal); writing‐original draft (equal); writing‐review & editing (equal). **Haitao Xu:** Data curation (equal); formal analysis (equal); investigation (equal); methodology (equal); validation (equal). **Wenfei Zhang:** Data curation (equal); formal analysis (equal); investigation (equal). **zhibiao chen:** conceptualization (equal); funding acquisition (equal); project administration (equal); supervision (equal); writing‐review & editing (equal). **Wenlan Li:** Conceptualization (equal); resources (equal); supervision (equal); writing‐review & editing (equal). **Wei Ke:** Conceptualization (equal); resources (equal); supervision (equal); writing‐review & editing (equal).

## Data Availability

The data used to support the findings of this study are available from the corresponding author upon request.
